# Light and Displacement Compensation-Based iPPG for Heart-Rate Measurement in Complex Detection Conditions

**DOI:** 10.3390/s24113346

**Published:** 2024-05-23

**Authors:** Shubo Bi, Haipeng Wang, Shuaishuai Zhang

**Affiliations:** 1 School of Intelligent Manufacturing, Jiangsu College of Engineering and Technology, Nantong 226006, China; wanghai463100@126.com; 2 Department of Precision Mechanical Engineering, Shanghai University, Shanghai 200444, China; zshuai@shu.edu.cn

**Keywords:** heart-rate measurement, iPPG, light compensation, displacement compensation

## Abstract

A light and displacement-compensation-based iPPG algorithm is proposed in this paper for heart-rate measurement in complex detection conditions. Two compensation sub-algorithms, including light compensation and displacement compensation, are designed and integrated into the iPPG algorithm for more accurate heart-rate measurement. In the light-compensation sub-algorithm, the measurement deviation caused by the ambient light change is compensated by the mean filter-based light adjustment strategy. In the displacement-compensation sub-algorithm, the measurement deviation caused by the subject motion is compensated by the optical flow-based displacement calculation strategy. A series of heart-rate measurement experiments are conducted to verify the effectiveness of the proposed method. Compared with conventional iPPG, the average measurement accuracy increases by 3.8% under different detection distances and 5.0% under different light intensities.

## 1. Introduction

Heart rate represents the number of heartbeats per minute (beat per minute, bpm), which is an effective indicator of human health [1]. There exists a high requirement for heart-rate measurement in multiple tasks such as household healthcare [2,3], medical treatment [4], and search and rescue [5]. Conventionally, the heart-rate measurement requires the subject to be equipped with contact measuring equipment. However, the subject cannot carry contact measurement devices in certain special environmental and task conditions. In such cases, the heart rate should be measured in a non-contact [6] way.

A series of sensors, including microwave radars, pressure sensors, and near-infrared cameras, can be used for non-contact heart-rate measurement. A wave of a fixed frequency shining on the object will produce a reflected wave of the same frequency. When the wave shines on the human heart (fixed-frequency vibration), a fixed-frequency mutation will be produced on the reflected wave according to the Doppler effect. In paper [7], a low power microwave radar is used to irradiate the human body, and the heart rate is measured by measuring the frequency mutation of the reflected wave. In paper [8], the pressure sensor is pasted onto the human body. The pressure sensor data will produce periodic fluctuations in the human heartbeat. The human heart rate can be calculated by analysis of the data fluctuations. The blood vessel pressure of human skin will change periodically when the human is in the heartbeat process. The temperature will also change with the same period. Paper [9,10] use a near-infrared camera to irradiate the human body and measure the human heart rate by measuring the temperature change period in a specific area. However, such sensors are not universal enough, which affects their popularization to some extent. By contrast, RGB video analysis-based heart-rate measurement has been widely used in recent years. Research [11] shows that RGB video analysis-based heart-rate measurement has the same accuracy as professional measurement equipment (such as radar).

iPPG (imaging Photoplethysmography) is the current mainstream RGB video analysis-based non-contact heart-rate measurement method [12,13,14]. In the method, exposed human skin images are captured and analyzed for pulse signal measurement. The pulse rate is equivalent to the heart rate. However, there still exist large differences in the signal-to-noise ratio (SNR) [15] by different detection and calculation strategies. The main reason for this is that different parts of the human body have different reflexes to ambient light, and the same area of the human body has different reflect abilities from different light spectrums [16,17,18]. In current studies, human facial [19,20] images are usually used for heart-rate measurement. In paper [21], specific spectral components are added to ambient light to improve the SNR. Another image registration algorithm is proposed in [22] to increase the SNR.

In paper [23], independent component analysis (ICA) was directly used to analyze the ambient reflected light of the R, G, and B channels, respectively. The method is simple but not robust enough to the subject motion, and the relationship between signals of different channels is unclear. Another principal component analysis (PCA) was proposed [24] to extract signals from the three channels. Compared with ICA, PCA can effectively reduce calculation time. In paper [25], the facial region was first divided into four sub-regions, and then the underlying signal set was obtained by the blind source separation of signals from different human skin regions. The heart-rate signal was recovered by spectral clustering of the underlying signal set. Based on this idea, a multi-objective optimization strategy was proposed [26] for signal selection in the blind source separation process. More accurate measurement results can be obtained based on this strategy.

The above research can achieve high measurement accuracy under ideal conditions. However, the accuracy will be significantly reduced when the ambient light changes or the subject moves. Paper [27] establishes two orthogonal chrominance signals based on the skin reflection model and uses the difference between the chrominance signals to cancel the specular reflection component to obtain a more accurate heart rate. Subsequently, the team proposed the CHROME (Chrominance-Based) [28] method and the color space normalization [29] method in turn to solve the interference caused by motion artifacts. In paper [30], joint blind source separation and ensemble empirical mode decomposition were used to reduce the interference caused by illumination. In paper [31], the image jitter is corrected based on a multi-task convolutional neural network, and the heart-rate signal is denoised by empirical mode decomposition and permutation entropy. Paper [32] proposes a gray level compensation algorithm to compensate for the change of gray level caused by the ambient light.

Based on the above methods, accurate heart-rate measurement can be achieved under small ambient light or displacement changes. However, when there is a relatively large change in the ambient light or the subject’s motion, the accuracy of the iPPG-based algorithm will be greatly disturbed. Based on this problem, a light and displacement-compensation-based iPPG algorithm is proposed in this paper. The method takes ordinary RGB video data as input to achieve the accurate measurement of human heart rate. Two sub-algorithms are designed and integrated into the conventional iPPG algorithm: the light-compensation sub-algorithm and the displacement-compensation sub-algorithm. The proposed method can better adapt to complex detection conditions. Firstly, the measurement deviation caused by the ambient light change is compensated by a light compensator. Secondly, the measurement deviation caused by the subject motion is compensated by a displacement compensator. The optical flow method [33,34] is used for the displacement calculation. A series of experiments in different detection conditions are conducted to verify the effectiveness of the proposed method. The experimental results show that the heart-rate measurement method proposed in this paper can effectively improve the accuracy of heart-rate measurement.

The rest of this paper is organized as follows: Section 2 details the basic procedure of the proposed method. The four phases of the proposed method are detailed in the later four sections (Section 3, Section 4, Section 5, and Section 6), respectively. Experiments are conducted in Section 7. Section 8 concludes the paper.

## 2. Overview of the Heart-Rate Measurement

**Figure 1:**
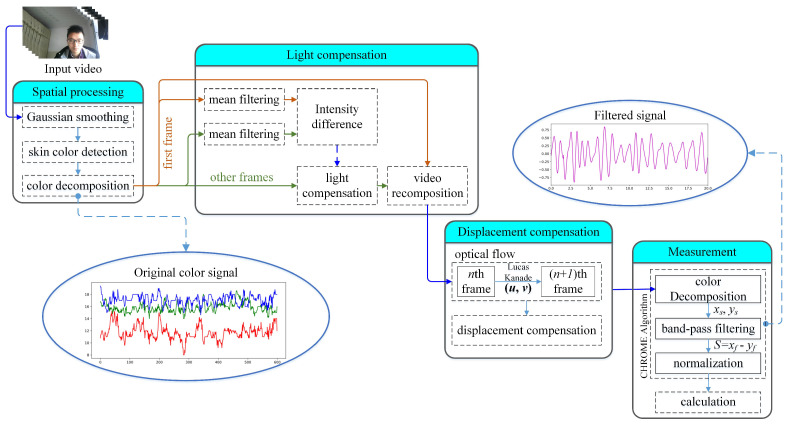
Heart-rate measurement flow chart.

Shown in Figure 1 is the heart-rate measurement flow chart. The method takes RGB video as input. The RGB video frame rate should be no less than 30 fps (frames per second), and the duration time should be no less than 20 s.

The whole process consists of four stages. Firstly, a series of spatial processing, including Gaussian smoothing, skin-color detection, and color decomposition, are conducted. Secondly, a light-compensation algorithm is proposed to compensate for the skin-reflect light changes caused by the ambient light change. The skin-reflect lights of the R, G, and B channels are compensated, respectively. Thirdly, a displacement-compensation algorithm is designed to compensate for the light intensity changes caused by the subject’s motion. The Lucas–Kanade(LK) optical flow algorithm is used for the subject displacement calculation. Finally, a band-pass filter is embedded in the CHROME algorithm for heart-rate measurement.

## 3. Spatial Processing

### 3.1. Gaussian Smoothing

In spatial processing, the Gaussian smoothing is first applied to all the frames to reduce image noise. The Gaussian function is defined as:

(1) $$G\left(x,y\right)=\frac{1}{2\pi \sigma^{2}}e^{-\frac{x^{2}+y^{2}}{2\sigma^{2}}},$$

where σ is the standard deviation, which is set to 1 in this paper. The intensity value of each pixel is updated to the average intensity value of itself and the nearby elements multiplied by a certain weight, *w*.

(2) $$I^{\prime }\left(x,y\right)=\frac{\sum_{i=0}^{4}\sum_{j=0}^{4}w_{ij}\times I\left(x+i-2,y+j-2\right)}{25},$$

where (x,y) is the initial intensity value of pixel (x,y) and $I^{\prime }\left(x,y\right)$ is the updated intensity value of I(x,y).

(3) $$w=\left[\begin{array}{ccccc} 0.003 & 0.013 & 0.022 & 0.013 & 0.003\\ 0.013 & 0.059 & 0.097 & 0.059 & 0.013\\ 0.022 & 0.097 & 0.159 & 0.097 & 0.022\\ 0.013 & 0.059 & 0.097 & 0.059 & 0.013\\ 0.003 & 0.013 & 0.022 & 0.013 & 0.003 \end{array}\right]$$

### 3.2. Skin-Color Detection Color Decomposition

*H* component of the HSV (Hue, Saturation, Value) color space and Cb and Cr components of the YCbCr space are selected for skin-color detection. The following constraints are designed:

(4) $$\left\{\begin{array}{c} 1\leq H\leq 23\\ 77\leq Cb\leq 127\\ 133\leq Cr\leq 173 \end{array}\right.$$

**Figure 2:**
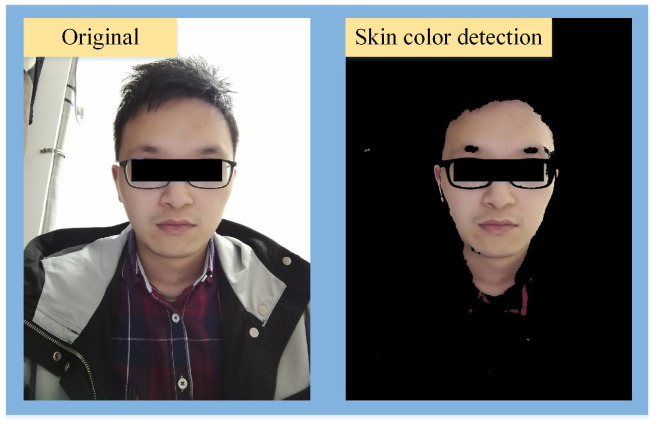
Result of skin-color detection.

Shown in Figure 2 is the skin detection result. It can be seen that the human facial region is successfully distinguished from the other areas. Finally, all the pixels in all the frames are decomposed into the red, green, and blue components, respectively.

(5) I(x,y)∈{R(i,j),G(i,j),B(i,j)},

where R(x,y), G(x,y), and B(x,y) are the red, green, and blue component of pixel (x,y), respectively.

## 4. Light Compensation

### 4.1. Light-Compensation Algorithm

A light compensator (see Algorithm 1) is proposed to compensate for the pixel intensity change caused by the ambient light change. Shown in the above algorithm is the process of the light-compensation algorithm. In the algorithm, the pixel intensities of the R, G, and B channels are compensated independently. The light-compensation algorithm assumes that the mean intensity value of any pixel with a fixed size is constant. Firstly, a mean filter with a window size of 2a×2b is applied to all the frames:

(6) $$g_{n}\left(i,j\right)=\frac{\sum_{p=-a}^{a}\sum_{q=-b}^{b}I_{t}^{\prime }\left(i+p,j+q\right)}{2a\times 2b},t\in \left[1,N\right],$$

where $g_{n}\left(i,j\right)$ is the mean pixel intensity of the area with the size of 2a×2b, which is constant to *C* (the constant). The center coordinates of the area is (i,j); *n* is the order of frames.

(7) $$g_{n}\left(i,j\right)\in \left\{R_{n}\left(i,j\right),G_{n}\left(i,j\right),B_{n}\left(i,j\right)\right\}$$

**Algorithm 1:** Light-compensation sub-algorithm**Input:** *R* = {*R*_1_, …, *R_N_*}, *G* = {*G*_1_, …, *G_N_*}, *B* = {*B*_1_, …, *B_N_*};/ / *N* represents the number of frames**Output:** $R*=\{R_{1}^{*},\ldots ,R_{N}^{*}\},G*=\{G_{1}^{*},\ldots ,G_{N}^{*}\},B*=\{B_{1}^{*},\ldots ,B_{N}^{*}\};$

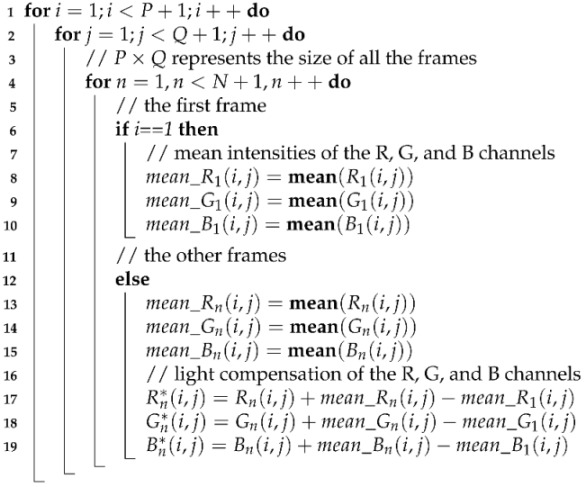



The intensities of each pixel in each frame are then added with the difference between $g_{n}\left(i,j\right)$ and $g_{1}\left(i,j\right)$.

(8) $$I_{n}^{*}\left(i,j\right)=I_{n}^{\prime }\left(i,j\right)+g_{n}\left(i,j\right)-g_{1}\left(i,j\right),$$

where $I_{n}^{*}\left(i,j\right)$ is the compensated light intensities of pixel (i,j).

### 4.2. Results of Light Compensation

**Figure 3:**
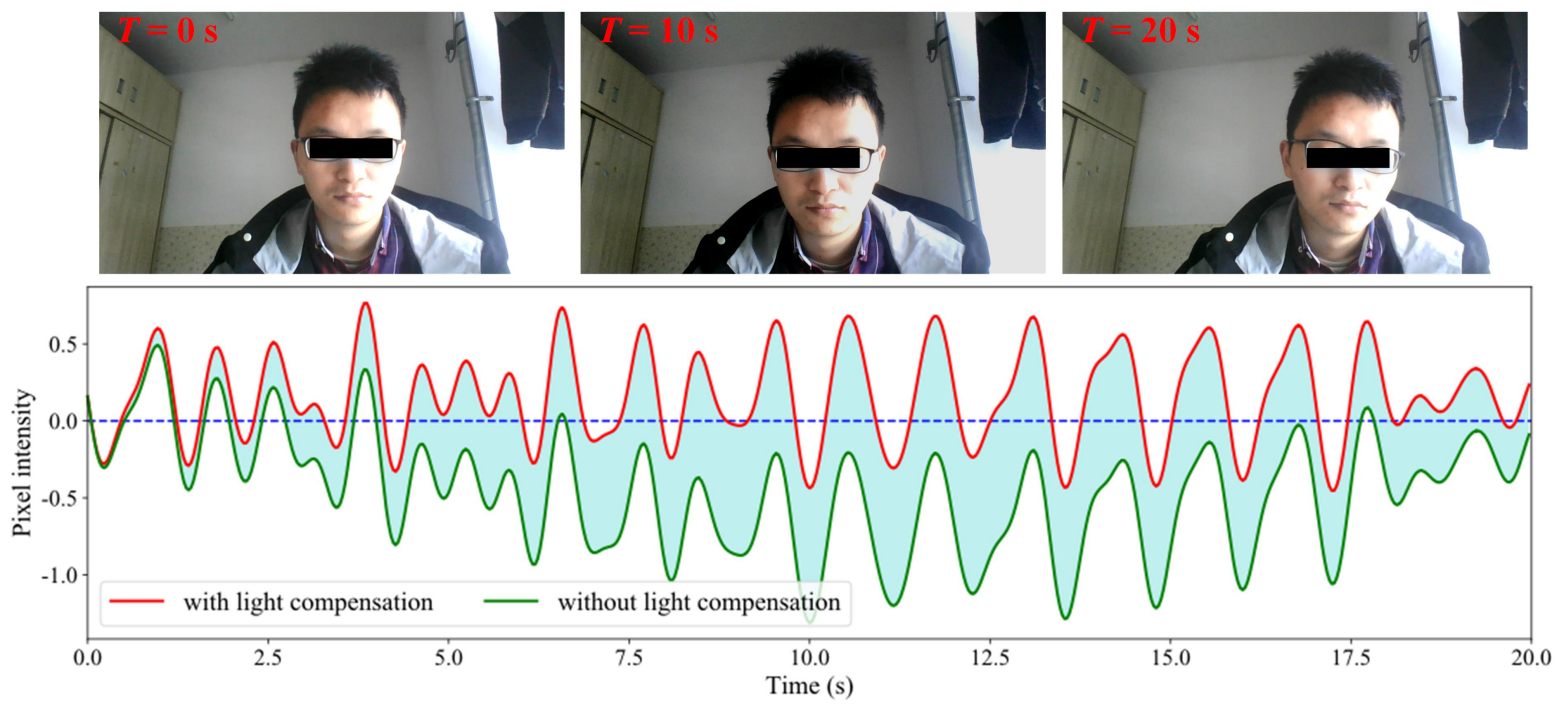
Results of light compensation.

Shown in Figure 3 is the light-compensation result. The upper shows the snapshots of the RGB video (20 s), and the lower shows the filtered curves. The red curve and green curve represent the pixel intensities with and without light compensation, respectively. The shaded area between the two curves represents the compensation values. It can be seen from the figure that the filtered curve has a large deviation relative to the initial state without light compensation. In the compensated curve, the deviation is eliminated.

## 5. Displacement Compensation

The LK algorithm is first used to evaluate the specific pixel’s displacement between any adjacent frames, and then the displacement is compensated for each pixel on each frame. In the LK algorithm, three typical assumptions are first introduced:

***Constant Brightness:*** Pixels of the target image in the scene appear to be unchanged as they move from frame to frame.

***Temporal Persistence (Small Movement):*** The motion of the objects on the image varies slowly over time.

***Spatial Consistency:*** Adjacent points on the same surface in the scene have similar motion and are projected relatively close to the image plane.

Suppose I(x,y,t) is the pixel value of the pixel point (x,y) at time *t*. After time dt, the subject’s specific point at pixel coordinate (x,y) moves to (x+dx,y+dy). According to the first assumption, there is:

(9) I(x,y,t)=I(x+dx,y+dy,t+dt).

According to the second assumption, the motion of the subjects on the image is small. Hence, the function I(x+dx,y+dy,t+dt) can be expanded at (x,y,t) using Taylor’s formula.

(10) $$I\left(x+dx,y+dy,t+dt\right)=I\left(x,y,t\right)+\frac{\partial I}{\partial x}+\frac{\partial I}{\partial y}+\frac{\partial I}{\partial t}+\epsilon ,$$

where ϵ represents the higher-order remainder of Taylor’s formula, which can be ignored. Therefore, there is:

(11) $$\frac{\partial I}{\partial x}+\frac{\partial I}{\partial y}+\frac{\partial I}{\partial t}=0.$$

Which is equivalent to:

(12) $$\frac{\partial I}{\partial x}\frac{\partial x}{\partial t}+\frac{\partial I}{\partial y}\frac{\partial y}{\partial t}+\frac{\partial I}{\partial t}=0,$$

where $\left(\frac{\partial x}{\partial t},\frac{\partial y}{\partial t}\right)=\left(u,v\right)$ is the optical flow of the pixel to be resolved. The velocity components along the *x* and *y* directions are denoted as u and v, respectively. The above formula can be written as:

(13) $$\left[\begin{array}{cc} I_{x} & I_{y} \end{array}\right]\left[\begin{array}{c} u\\ v \end{array}\right]=-I_{t}.$$

This formula shows that the temporal pixel value differential at the same coordinate position is the production of the spatial pixel value differential and the velocity relative to the observer at that position. However, as the solution of the above binary linear equation is not unique, other constraints are required.

According to the third assumption, it can be assumed that within a window of size m×m, the optical flow of the image is a constant value. Hence, the following equation can be obtained:

(14) $$\left[\begin{array}{cc} I_{x1} & I_{y1}\\ I_{x2} & I_{y2}\\ \vdots & \vdots \\ I_{xm} & I_{ym} \end{array}\right]=-\left[\begin{array}{c} I_{t1}\\ I_{t2}\\ \vdots \\ I_{tm} \end{array}\right]\Leftrightarrow A\vec{V}=-b.$$

The final result can be calculated by:

(15) $$\left[\begin{array}{c} u\\ v \end{array}\right]={\left[\begin{array}{cc} \sum_{i=1}^{n}I_{xi}^{2} & \sum_{i=1}^{n}I_{xi}I_{yi}\\ \sum_{i=1}^{n}I_{xi}I_{yi} & \sum_{i=1}^{n}I_{yi}^{2} \end{array}\right]}^{-1}\left[\begin{array}{c} -\sum_{i=1}^{n}I_{xi}I_{ti}\\ -\sum_{i=1}^{n}I_{yi}I_{ti} \end{array}\right].$$

**Figure 4:**
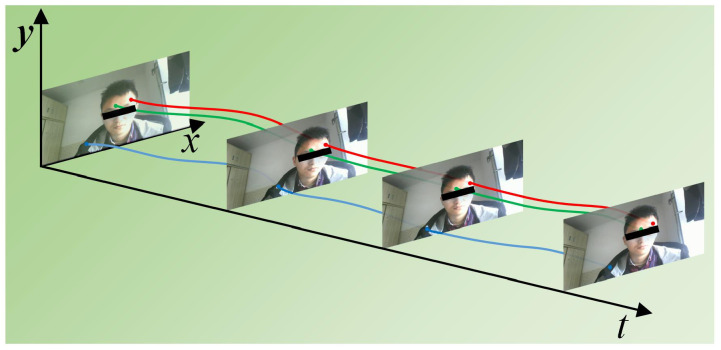
Displacements of specific pixels in the video.

Displacement measurement experiments are conducted to verify the effectiveness of the algorithm. The results are shown in Figure 4. Select three random points on the human body in the video and track the movement. The trajectory curves are shown as the color lines. It can be seen that the optical flow method-based displacement measurement algorithm can accurately track the coordinates of specific pixels when the subject moves, then the light intensity change of a specific point on the video can be obtained.

## 6. Heart-Rate Measurement

### 6.1. Design of the Filter

The human heart rate ranges approximately from 1.0 Hz to 1.667 Hz. In this paper, a Butterworth band-pass filter is used to filter out the high-frequency and low-frequency noises. The magnitude of the frequency response (first order) is given by

(16) $$H\left(j\omega \right)=\frac{j\omega \tau_{1}}{1+j\omega \tau_{1}}\frac{1}{j\omega \tau_{2}}$$

(17) $$\left[\begin{array}{c} W_{1},W_{2} \end{array}\right]=\left[\begin{array}{c} \frac{1}{\tau_{1}},\frac{1}{\tau_{2}} \end{array}\right],$$

where the passband is $\left[W_{1},W_{2}\right]$. The filter performance is influenced by two aspects: the order of the filter and the range of the filter passband.

**Figure 5:**
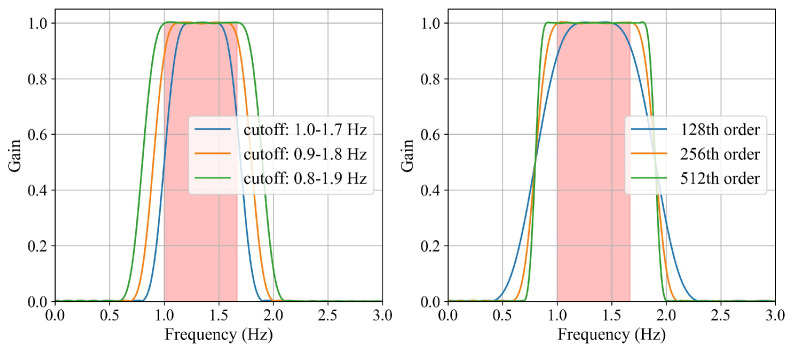
The frequency response of the band-pass filter.

Shown in Figure 5 is the frequency response of the band-pass filter. The red zone represents the range of the human heart rate. The order is 256 on the left curve, and the passband ranges from 0.8 to 1.9 on the right curve. It can be seen that the stopband falls faster with the increase of the filter order, and the filter pass width is widened as the passband widens. Based on the above analysis, a 256th-order band-pass filter with a passband from 0.8 Hz to 1.9 Hz is utilized to let through the maximum frequency of human heart rate without attenuation.

### 6.2. Color Decomposition

This paper uses the CHROME algorithm to measure the robustness. First, decompose the light as follows:

(18) $$C_{k}=I_{C_{k}}\left(\rho_{C_{dc}}+\rho_{C_{k}}+S_{k}\right),$$

where $C_{k}$ is the *C* channel intensity of a particular pixel in graph *k*. C∈{R,G,B}. $I_{C_{k}}$ represents the intensity of $C_{k}$ in the camera’s exposure stage. $\rho_{C_{dc}}$ represents the fixed coefficient of reflection of light from the skin surface, which is fixed but different in different channels: $\rho_{R_{dc}}>\rho_{G_{dc}}>\rho_{B_{dc}}$. $\rho_{C_{k}}$ represents the component of dynamic disturbance caused by the pulse. $S_{k}$ represents the specular reflection component due to motion, which is the same in different channels.

The CHROME algorithm combines the signals of the R, G, and B channels in different proportions to eliminate the static component and the additional specular reflection component and outputs the pulsating diffuse reflection component. The combination mode is as follows:

(19) $$\left\{\begin{array}{c} X_{s}=3R\left(n\right)-2G\left(n\right)\\ Y_{s}=1.5R\left(n\right)+G\left(n\right)-1.5B\left(n\right) \end{array},\right.$$

where R(n), G(n), and B(n) are the normalized signals of the R, G, and B channel. $X_{s}$ and $Y_{s}$ are filtered by the band-pass filter, respectively, after the filters $X_{f}$ and $Y_{f}$ are obtained. Finally, the heart-rate signal is formed by the following formula:

(20) $$S=X_{f}-\alpha Y_{f}.$$

**Figure 6:**
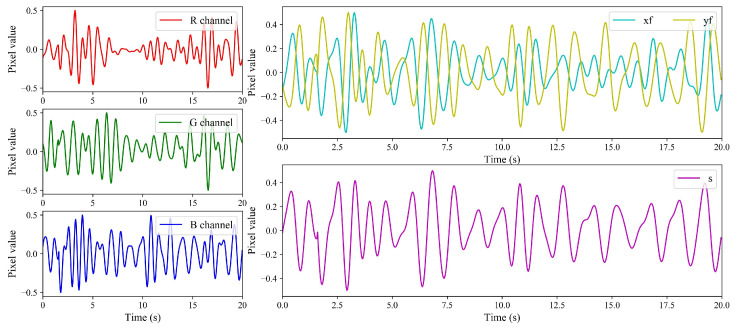
Color decomposition of one specific pixel.

The filtering results are shown in Figure 6. The three figures on the left are the partial filtering results of the R, G, and B channels, respectively. The figure on the upper right shows the result of $X_{f}$ and $Y_{f}$, and the figure on the lower right shows the value of *S*. As can be seen from the figure, the filtered data of the R, G, and B channels fluctuate greatly, which makes it difficult to filter effective heartbeat signals from them. The fluctuation of *S* obtained by $X_{f}$ and $Y_{f}$ is relatively uniform. Follow-up heart-rate data extraction can be performed.

### 6.3. Heart-Rate Measurement


(21) $$cycle=\frac{\sum_{i=1}^{n}cycle_{i}}{n}$$

The time interval between any two adjacent peaks in the pixel value curve is regarded as a heartbeat cycle, and the average of all heartbeat cycles is considered as the heart rate of the human. As shown in Equation (21), $cycle_{i}$ is the ith heartbeat cycle, and cycle is the average heart rate of the human.

**Figure 7:**
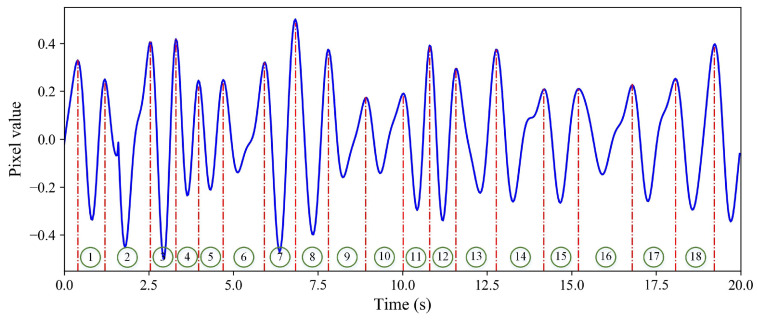
Pixel value of one specific pixel.

Shown in Figure 7 is the pixel value of a specific point on the RGB video. The horizontal coordinate represents the time, and the vertical coordinate represents the pixel value after standardization. The data have been filtered and standardized. The processed data values are between −0.5 and 0.5. It can be seen from the figure that the curve has obvious periodic characteristics. The height of each wave crest and trough are not the same; the main reason for this is that there is a certain difference in the heartbeat amplitude of each human. The time between the two peaks (or troughs) in the curve is the heartbeat cycle of the human hour. To ensure measurement accuracy, a 20-s video is collected to calculate the average heartbeat cycle. It can be seen that a total of 18 complete heartbeat cycles exist in the curve. The mean cycle time is 18.82 s. Hence, the heart rate is 60/(18.82/18) = 57.4 bpm.

## 7. Experiment

**Figure 8:**
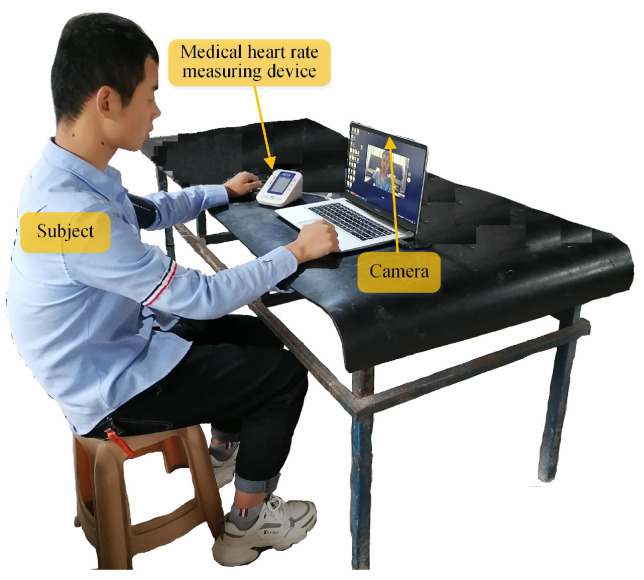
The experimental scene.

The experimental scene is shown in Figure 8. A built-in camera of a laptop computer is used for the subject’s facial videos collection. The subject sits in front of the laptop with their face facing the camera of the laptop to facilitate image acquisition. The size of the frame is 1920 × 1080 pixels, and the frame rate is 30 fps. Video capture duration is no less than 20 s. The subjects are asked to remain as still as possible during the data collection. A total of five subjects participated in the data collection. Meanwhile, a medical heart-rate measurement device is used as the control group. The measurement device is fixed on the human’s arm at the same horizontal height as the human’s heart. The data and images of the control group are collected at the same time. Each subject captured five videos in each set of experimental conditions (specific light and detection distance). The real-time heart rate of the subjects during the collection of each video is recorded at the same time.

**Figure 9:**
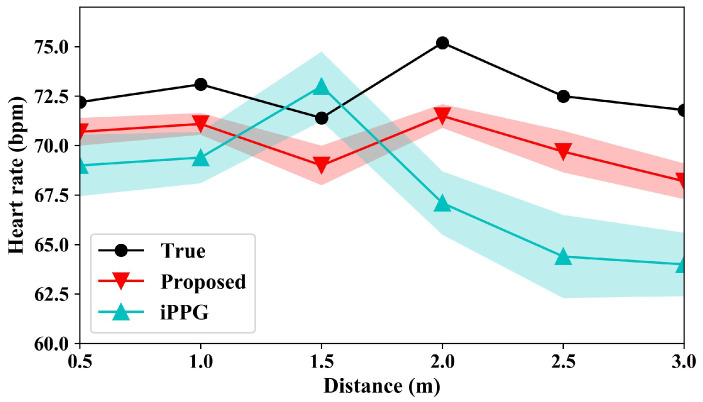
Heart-rate measurement results under various human–camera distances.

Shown in Figure 9 are the heart-rate measurement results under various human–camera distances. The light intensity is approximately 300 lux in this group. It can be seen that the subjects’ heartbeat fluctuates at different distance conditions, but the overall change is small. The heart rate calculated by the proposed method is less than the actual heart rate. The main reason for this is that part of the human heartbeat is light and filtered by filters. There exists a large deviation in heart-rate measurement by all the methods. The average measurement accuracy is 7.4% by the traditional iPPG-based algorithm, and the average measurement accuracy increases to 3.6% with the proposed method. The results show that the proposed method can increase the heart-rate measurement accuracy at any human–camera distances.

**Figure 10:**
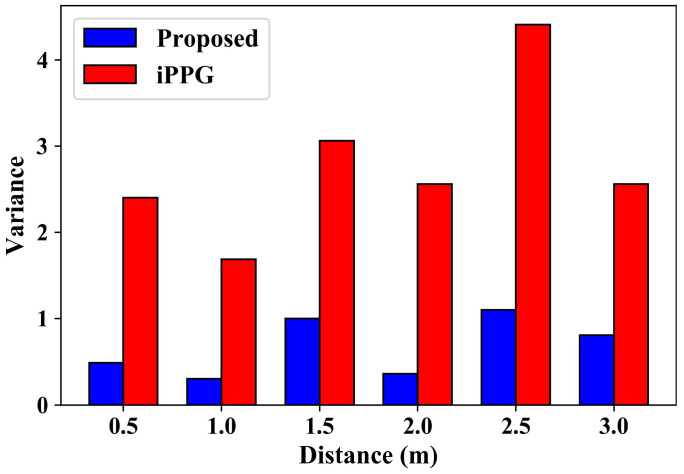
Variances under various human–camera distances.

Figure 10 show the variances under various human–camera distances. The greater the variance, the greater the fluctuation and the worse the stability of the measurement results. It can be seen from the results that the measurement results using the conventional IPPG-based algorithm have a large fluctuation, which is much higher than that of the proposed method. The data fluctuation is suppressed to a large extent using the proposed method. At the same time, it can be seen that the human–camera distance has little influence on the measurement accuracy under the two methods. This means that the method proposed can achieve high-precision heart-rate measurements at a distance of less than 3 m. Generally, the average measurement accuracy is improved by 3.8% at different distances by the proposed compensation algorithm.

**Figure 11:**
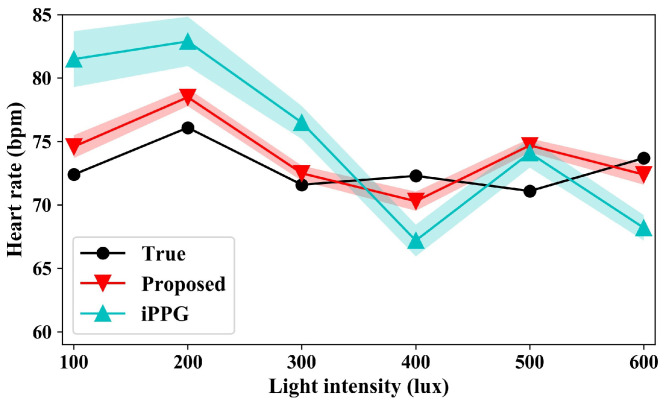
Heart-rate measurement results under different light intensities.

Shown in Figure 11 are the heart-rate measurement results under different light intensities. The human–camera distance in this group is 1.0 m. It can be seen from the figure that the subjects’ heart rates fluctuate to some extent under different lighting conditions, but the overall change was small. At the same time, with the increase in light intensity the difference between the measured results and the real heart rate gradually decreased. The main reason for this is that there exists more noise under low light conditions, and some image noise cannot be effectively filtered out, which results in a high heart-rate measurement. The environmental noise is effectively filtered with the increase of light intensities. On the contrary, the weaker heartbeat of the human part was filtered out by the filter, and the measured value was slightly lower than the true value. On the whole, the average measurement accuracy is 7.8% without compensation, and the average measurement accuracy increases to 2.8% by the proposed method.

**Figure 12:**
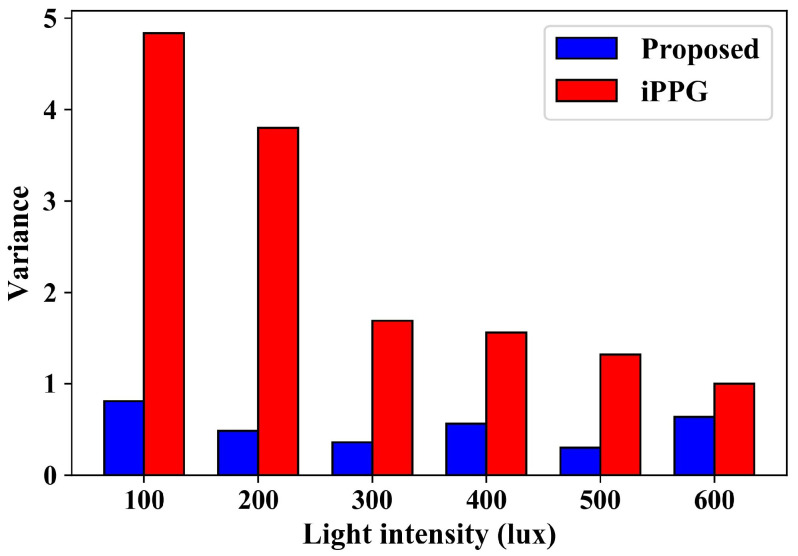
Variances under different light intensities.

Figure 12 shows the variances under different light intensities. It can also be seen that large variances exist in heart-rate measurement by the iPPG-based algorithm. The variance is greatly suppressed by the proposed method. In the case of low light intensity, the algorithm based on iPPG is more sensitive to light, and the variance is larger under low-light conditions, indicating that the measurement results fluctuate greatly under low-light conditions. However, the proposed method can guarantee a low variance under any illumination conditions, indicating a good consistency of measurement results.

## 8. Discussion and Conclusions

Aimed at the problem of the low robustness of current heart-rate measurement methods in complex detection conditions, a light and displacement-compensation-based iPPG algorithm is proposed in this paper for heart-rate measurement. The method consists of four main procedures: spatial processing, light compensation, displacement compensation, and measurement. Only an RGB video with a human face is required in this method.

The input video is first processed by a Gaussian fuzzy filter, and then the video is decomposed frame by frame. The light-compensation module is used to compensate for the influence caused by ambient light on the measurement results. Displacement compensation is used to compensate for the influence caused by the subject’s movement in a small range. To measure the displacement of the humans, a displacement measurement method based on the optical flow method is introduced in this study. The measurement deviation caused by the ambient light change and subject motion is compensated by the two compensation sub-algorithms. In the final measurement stage, the CHROME algorithm is first used to decompose and reassemble the image, then the band-pass filter is used to filter the input data frame by frame and pixel by pixel, and the clutter outside the human heart-rate range is filtered out. Finally, the heart rate is calculated.

A series of experiments in different detection conditions (various light intensities and various human–camera distances) are conducted to verify the effectiveness of the proposed method. Compared with traditional methods, the proposed method can achieve higher measurement accuracy and lower measurement variance. Specifically, the following conclusions are drawn:

(1) The average measurement accuracy is improved by 3.8% by the proposed compensation algorithm under different distance conditions.

(2) The average measurement accuracy is improved by 5.0% by the proposed compensation algorithm under different light intensities.

(3) To achieve a higher accuracy of heart-rate measurement, the detection distance should not exceed 2 m and the lighting intensity should not be less than 200 lux.

The proposed method can be used for heart-rate measurement under small movements of the human body. However, when the motion range of the human body is large, the processing capacity of the optical flow method makes it unable to accurately measure the heart rate. In future work, the key points of the face should be identified under large-scale human movement, and then the heart-rate measurement could also be realized under large-scale human movement.

## Data Availability

Raw data underlying the results presented in this paper are available from the authors upon reasonable request.

## Abbreviations

The following abbreviations are used in this manuscript:

bpm	beat per minute
iPPG	imaging Photoplethysmography
SNR	signal-to-noise ratio
ICA	independent component analysis
PCA	principal component analysis
CHROME	Chrominance-Based
fps	frames per second
LK	Lucas–Kanade
